# Prognostic Impact of Anemia and Hemoglobin Levels in Unselected Patients Undergoing Coronary Angiography

**DOI:** 10.3390/jcm13206088

**Published:** 2024-10-12

**Authors:** Philipp Steinke, Tobias Schupp, Lasse Kuhn, Mohammad Abumayyaleh, Kathrin Weidner, Thomas Bertsch, Alexander Schmitt, Mahboubeh Jannesari, Fabian Siegel, Daniel Duerschmied, Michael Behnes, Ibrahim Akin

**Affiliations:** 1 Department of Cardiology, Angiology, Haemostaseology and Medical Intensive Care, University Medical Centre Mannheim, Medical Faculty Mannheim, Heidelberg University, 68167 Mannheim, Germany; 2 Institute of Clinical Chemistry, Laboratory Medicine and Transfusion Medicine, Nuremberg General Hospital, Paracelsus Medical University, 90419 Nuremberg, Germany; 3 Department of Biomedical Informatics, Center for Preventive Medicine and Digital Health (CPD), Medical Faculty Mannheim, Heidelberg University, 68167 Mannheim, Germany

**Keywords:** anemia, hemoglobin, coronary artery disease, coronary angiography, prognosis

## Abstract

**Background/Objectives**: This study investigates the prevalence and prognostic impact of concomitant anemia in unselected patients undergoing invasive coronary angiography (CA). The spectrum of patients undergoing CA has significantly changed during the past decades, related to ongoing demographic changes and improved treatment strategies for patients with cardiovascular disease. **Methods**: Consecutive patients undergoing invasive CA from 2016 to 2022 were retrospectively included at one institution. Patients with anemia (i.e., hemoglobin < 13.0 g/dL for males and <12.0 g/dL for females) were compared with patients without anemia (i.e., nonanemics). The primary endpoint was rehospitalization for heart failure (HF) at 36 months. Secondary endpoints comprised the risk of rehospitalization for acute myocardial infarction (AMI) and coronary revascularization. Statistical analyses included Kaplan–Meier, multivariable Cox proportional regression analyses, and propensity score matching. **Results**: From 2016 to 2022, 7645 patients undergoing CA were included with a median hemoglobin level of 13.2 g/dL. Anemics had a higher prevalence of coronary artery disease (CAD) (76.3% vs. 74.8%; *p* = 0.001), alongside an increased need for percutaneous coronary intervention (PCI) (45.3% vs. 41.5%; *p* = 0.001). At 36 months, the risk of rehospitalization for HF was higher in anemic patients (27.4% vs. 18.4%; *p* = 0.001; HR = 1.583; 95% CI 1.432–1.750; *p* = 0.001), which was still evident after multivariable adjustment (HR = 1.164; 95% CI 1.039–1.304; *p* = 0.009) and propensity score matching (HR = 1.137; 95% CI 1.006–1.286; *p* = 0.040). However, neither the risk of AMI (8.4% vs. 7.4%, *p* = 0.091) nor the risk of coronary revascularization at 36 months (8.0% vs. 8.5%, *p* = 0.447) was higher in anemic compared with nonanemic patients. **Conclusions**: In consecutive patients undergoing CA, concomitant anemia was independently associated with an increased risk of rehospitalization for HF, but not AMI or coronary revascularization. Patients with LVEF ≥ 35% and multivessel disease were especially susceptible to anemia-induced HF-related rehospitalization.

## 1. Introduction

According to the 2019 Global Burden of Disease Study, an estimated 197 million people suffered from coronary artery disease (CAD) in 2019 worldwide, with a corresponding prevalence of 2.7% [1]. This represents an increase of 63% since 2000 [1]. In 2019, 9.1 million deaths and 182 million disability-adjusted life years (DALYs) worldwide were directly attributed to CAD, which represents an increase of 2.6 million and 44 million, respectively, since 2000 [1]. Due to substantial improvements in treatment options over the past two decades, including improvements in percutaneous coronary intervention (PCI), coronary artery bypass graft (CABG) techniques, and pharmacotherapies, CAD-related mortality rates have substantially decreased [2,3].

Comorbidities in patients with CAD are widely recognized to have a significant impact on prognosis and CAD-related outcomes [4,5]. One of these comorbidities is anemia, which is prevalent in up to 71% of patients with CAD [6]. Anemia may lead to increased preload, decreased afterload, and activation of the sympathetic nervous system, which results in an increase in the heart rate and left ventricular hypertrophy [7,8]. Subsequently, anemia may increase myocardial oxygen demand and worsen cardiac ischemia [9]. Even in patients with nonsignificant coronary artery disease, acute anemia may lead to transient myocardial ischemia and acute myocardial infarction (AMI), specifically type II AMI [10]. Moreover, anemia has not only been suggested to be an independent risk factor for CAD but also an independent risk factor for major cardiovascular events, such as AMI and cardiovascular death [11].

However, studies investigating the prevalence and prognostic value of anemia in unselected patients undergoing coronary angiography (CA) are limited. This, along with ongoing demographic changes and improved treatment strategies for patients with cardiovascular disease, makes a precise characterization of patients undergoing CA necessary.

Therefore, the present study sought to investigate the prognostic impact of anemia, as well as different hemoglobin levels in unselected patients undergoing CA, using a large retrospective registry-based dataset.

## 2. Materials and Methods

### 2.1. Study Patients, Design, and Data Collection

For the present study, all consecutive patients undergoing CA at the University Medical Centre Mannheim (UMM), Germany, were included from January 2016 to August 2022. Patients were identified using Operation and Procedure Classification (OPS) codes. The local electronic hospital information system (SAP^®^, Walldorf, Germany) was used to retrospectively document all relevant clinical data related to the index event, including symptoms and diagnosis on admission, prior medical history, angiographic findings, and interventions, as well as medication on discharge. Patients who underwent recurrent CA were only included once.

The present study was derived from a retrospective single-center registry including consecutive patients undergoing CA, hospitalized at the University Medical Centre Mannheim (UMM), Germany (DRKS-ID: DRKS00034765). The registry was developed according to the principles of the Declaration of Helsinki and was approved by the medical ethics committee II of the Medical Faculty Mannheim, University of Heidelberg, Germany (ethics committee approval code: 2022-829).

### 2.2. Inclusion and Exclusion Criteria

For the present study, all consecutive patients ≥ 18 years of age undergoing invasive CA at one institution were included. CAs were performed by interventional cardiologists in accordance with current European guidelines [12]. CA operators were blinded to the final study analyses. For the present study, all source data of coronary angiographic examinations (imaging files) and reports were reassessed post hoc by two independent cardiologists. Patients without hemoglobin measurements on admission were excluded. No further exclusion criteria were applied for the present study.

### 2.3. Risk Stratification

The diagnosis of anemia was made according to the definition of the World Health Organization (WHO). Consequently, all men exhibiting a hemoglobin concentration < 13 g/dL and all women exhibiting a hemoglobin concentration < 12 g/dL, were diagnosed with anemia. Further risk stratification was performed stratified by hemoglobin decrease (per 1 g/dL). In patients with multiple hemoglobin measurements, risk stratification was performed according to the median hemoglobin level.

### 2.4. Study Endpoints

The primary endpoint was rehospitalization for heart failure (HF) at 36 months. Secondary endpoints comprised the risk of AMI and coronary revascularization at 36 months, as well as in-hospital all-cause mortality. All endpoints were defined using International Classification of Diseases (ICDs) codes at the University Medical Centre Mannheim (UMM), Germany.

### 2.5. Statistical Methods

Quantitative data is presented as mean ± standard error of the mean (SEM), median, and interquartile range (IQR), as well as ranges depending on the distribution of the data. They were compared using the Student’s *t*-test for normally distributed data or the Mann–Whitney U test for nonparametric data. Deviations from a Gaussian distribution were tested by the Kolmogorov–Smirnov test. Qualitative data is presented as absolute and relative frequencies and were compared using the Chi-square test or Fisher’s exact test, where appropriate. Kaplan–Meier analyses investigating the risk of HF-related rehospitalization, AMI, and coronary revascularization were performed including patients discharged alive. Univariable hazard ratios (HR) were given together with 95% confidence intervals (CIs). The prognostic impact of CAD etiology was thereafter investigated within multivariable Cox regression models using the “forward selection” option. Multivariable Cox regression analyses were performed within the entire study cohort, as well as in prespecified subgroups stratified by >75 and ≤75 years of age, presence of angina, (non-)ST-elevation myocardial infarction ((N)STEMI), and decompensated HF at index hospitalization, multivessel disease and left ventricular ejection fraction (LVEF), diabetes, glomerular filtration rate (GFR < 60 mL/min), and the presence or absence of malignancies. Multivariable Cox regression analyses were visualized using forest plots.

Due to the heterogeneous distribution of baseline characteristics and comorbidities within a registry of unselected consecutive patients, propensity score matching was performed to create more balanced subgroups and subsequently re-evaluate the prognostic impact of anemia in patients undergoing CA. Propensity score matching was applied for the comparison of patients with and without anemia, including the entire study cohort, along with the application of a nonparsimonious multivariable logistic regression model. Propensity scores were created according to the presence of the following independent variables: age, sex, diabetes mellitus, prior chronic obstructive pulmonary disorder (COPD), prior kidney insufficiency, prior malignancy, STEMI, NSTEMI, atrial fibrillation, acute decompensated HF, angina pectoris, resuscitation, valvular heart disease, LVEF, CAD type, GFR, white blood cells, platelets, and potassium levels. Based on the propensity score values generated by logistic regression, each anemic patient was matched with a patient without anemia with a similar propensity score value (accepted difference in propensity score value < 5%). Within the propensity-score-matched subgroup, the Kaplan–Meier method was applied, and univariable HRs were given together with 95% CIs. For all baseline characteristics, procedural, laboratory, and follow-up data, the standard mean differences were calculated.

Results of all statistical tests were considered significant for *p* ≤ 0.05. SPSS (Version 25, IBM, Armonk, NY, USA) was used for statistics.

## 3. Results

### 3.1. Study Population

From January 2016 until August 2022, 7691 patients underwent CA at the catheterization unit of the University Medical Centre Mannheim (UMM), Germany. A total of 46 patients without hemoglobin measurements were excluded. The final study cohort comprised 7645 patients undergoing CA, with a median hemoglobin level of 13.2 g/dL (IQR: 11.6–14.5 g/dL) (corresponding prevalence of anemia: 38.5%). Patients’ characteristics and comorbidities are outlined in Table 1. Patients with anemia were older (median age 75 vs. 66 years; *p* = 0.001), less likely to be males (63.1% vs. 66.3%, *p* = 0.004), and had a lower body mass index (BMI) (median: 26.5 vs. 27.8, *p* = 0.001) (Table 1). Regarding cardiovascular risk factors, anemics had a higher prevalence of diabetes mellitus (31.3% vs. 21.8%, *p* = 0.001), but a lower prevalence of arterial hypertension (82.2% vs. 87.1%, *p* = 0.001) and hyperlipidemia (26.8% vs. 41.1%, *p* = 0.001) compared with nonanemics. Additionally, patients with anemia had higher rates of prior stroke (1.1% vs. 0.6%, *p* = 0.035). (Table 1). Moreover, the rate of concomitant malignancy (10.0% vs. 3.2%, *p* = 0.001) was higher in anemics. Patients with anemia had significantly higher rates of moderately (35–44%) (18.0% vs. 11.7%; *p* = 0.001) and severely reduced LVEF (<35%) (18.6% vs. 13.0%; *p* = 0.001) in comparison to nonanemics (Table 1).

Accordingly, CAD was found more frequently in the anemic cohort (evidence of CAD: 74.8% vs. 66.3%; *p* = 0.001), specifically in those with higher rates of 3-vessel disease (36.1% vs. 24.9%; *p* = 0.001) (Table 2). As a result, PCI (45.3% vs. 41.5%, *p* = 0.001) and CABG (1.1% vs. 0.5%, *p* = 0.002) were more frequently performed in anemics. Regarding laboratory data, anemic patients had higher levels of C-reactive protein (CRP) (median 60 vs. 14 mg/L, *p* = 0.001) and amino-terminal pro-B-type natriuretic peptide (NT-proBNP) (median 3817 vs. 1064 pg/mL, *p* = 0.001) (Table 2).

### 3.2. Prognostic Value of Anemia in Patients Undergoing CA

**Figure 1:**
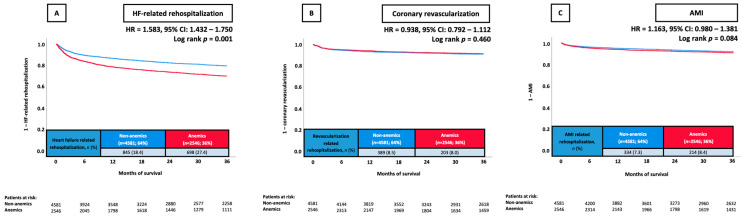
Prognostic impact of anemia in unselected patients on the risk of HF-related rehospitalization at 36 months (**A**), coronary revascularization at 36 months (**B**), and AMI at 36 months (**C**). AMI, acute myocardial infarction; CI, confidence interval; HF, heart failure; HR, hazard ratio.

At 36 months, the primary endpoint of rehospitalization for HF occurred in 27.4% of patients with anemia and in 18.4% without anemia. Anemics had a higher risk of HF-related rehospitalization at 36 months compared with non-anemics (HR = 1.583; 95% CI 1.432–1.750; *p* = 0.001) (Figure 1A).

**Figure 2:**
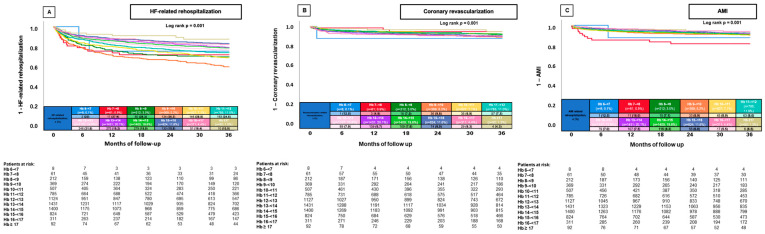
Prognostic impact of hemoglobin (stratified by 1 g/dL increase) in unselected patients on the risk of HF-related rehospitalization at 36 months (**A**), coronary revascularization at 36 months (**B**), and AMI at 36 months (**C**). AMI, acute myocardial infarction; HB, hemoglobin; HF, heart failure.

When further stratifying by hemoglobin levels (per 1 g/dL decrease), lower and higher hemoglobin levels were associated with a decreased risk of HF-related rehospitalization. Patients with hemoglobin levels of 9–<10 g/dL had the highest rates of HF-related rehospitalization (rehospitalization rate 36.4%, log-rank *p* = 0.001) (Figure 2A).

In contrast, no significant difference regarding the risk of rehospitalization for AMI (8.4% vs. 7.3%, *p* = 0.091) and coronary revascularization at 36 months (8.0% vs. 8.5%, *p* = 0.447) due to anemia was found (Figure 1B,C)

### 3.3. Multivariable Cox Regression and Propensity-Score-Matched Analyses

**Figure 3:**
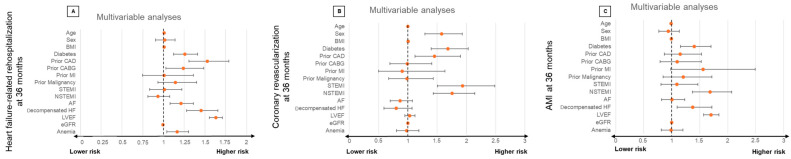
Forest plots displaying multivariable Cox regression analyses with regard to the risk of HF-related rehospitalization at 36 months (**A**), coronary revascularization at 36 months (**B**), and AMI at 36 months (**C**) within the entire study cohort. AF, atrial fibrillation; AMI, acute myocardial infarction; BMI, body mass index; CABG, coronary artery bypass grafting; CAD, coronary artery disease; eGFR, estimated glomerular filtration rate; HF, heart failure; LVEF, left ventricular ejection fraction; N(STEMI), non-ST elevation myocardial infarction.

Even after multivariable adjustment, the presence of concomitant anemia was still associated with an increased risk of HF-related rehospitalization (HR = 1.164; 95% CI: 1.039–1.304; *p* = 0.009) (Figure 3). Furthermore, higher age (HR = 1.009, 95% CI: 1.004–1.014; *p* = 0.001; per year increase), diabetes mellitus (HR = 1.258; 95% CI: 1.122–1.411; *p* = 0.001), prior CAD (HR = 1.529; 95% CI: 1.307–1.789; *p* = 0.001), atrial flutter (HR = 1.211; 95% CI: 1.080–1.359; *p* = 0.001), ADHF during index hospitalization (HR = 1.454; 95% CI: 1.277–1.656; *p* = 0.001) and LVEF < 35% (HR = 1.630; 95% CI: 1.554–1.710; *p* = 0.001) were associated with a higher risk of HF-related rehospitalization. In contrast, a higher GFR was associated with a lower risk (HR = 0.995; 95% CI: 0.992–0.997; *p* = 0.001; per 1 mL/min increase) (Figure 3).

**Figure 4:**
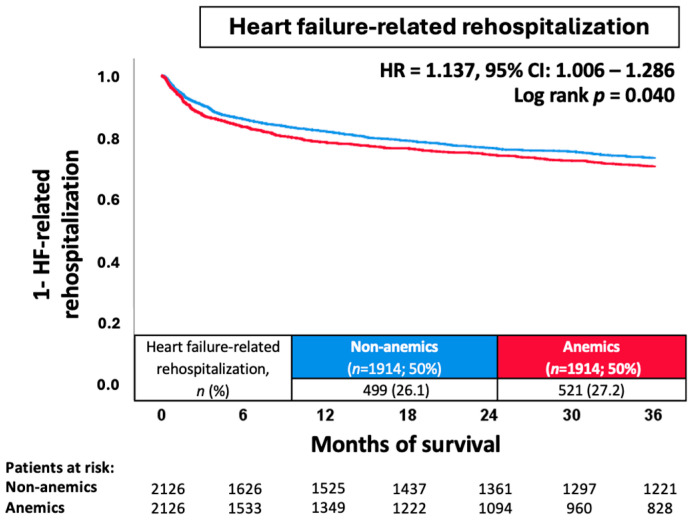
Prognostic impact of anemia after propensity score matching in unselected patients on the risk of HF-related rehospitalization at 36 months. CI, confidence interval; HF, heart failure; HR, hazard ratio.

Due to the heterogeneous distribution of baseline characteristics and comorbidities of patients with and without anemia, propensity score matching was performed to investigate the impact of anemia on more balanced subgroups. After propensity score matching (*n* = 1914 matched pairs), a significantly higher risk of HF-related rehospitalization was observed for anemic patients (27.2% vs. 26.1%; HR = 1.137; 95% CI 1.006–1.286; *p* = 0.040) (Figure 4).

### 3.4. Subgroup Analysis

**Figure 5:**
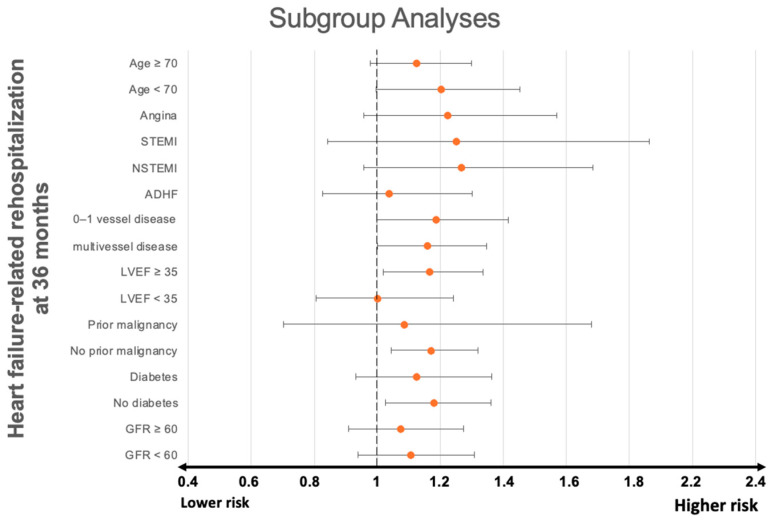
Forest plot displaying subgroup analyses investigating the prognostic impact of concomitant anemia with regard to the risk of HF-related rehospitalization at 36 months. ADHF, acute decompensated heart failure; GFR, glomerular filtration rate; LVEF, left ventricular ejection fraction; N(STEMI), non-ST elevation myocardial infarction.

The presence of anemia was also associated with a significantly higher risk of HF-related rehospitalization in patients presenting with multivessel disease (HR = 1.166, 95% CI: 1.018–1.336; *p* = 0.026), LVEF ≥ 35 (HR = 1.161, 95% CI: 1.000–1.347; *p* = 0.050), whereas anemia had no significant effect on the risk of 36-month HF-related rehospitalization in patients presenting with unstable angina (HR = 1.225, 95% CI: 0.956–1.569; *p* = 0.108), STEMI (HR = 1.252, 95% CI: 0.841–1.864; *p* = 0.269), NSTEMI (HR = 1.269; 95% CI: 0.957–1.685; *p* = 0.098), ADHF (HR = 1.037; 95% CI: 0.827–1.301; *p* = 0.754), prior malignancy (HR = 1.086, 95% CI: 0.702–1.68, *p* = 0.711), diabetes mellitus (HR = 1.127, 95% CI: 0.932–1.363, *p* = 0.219), or GFR < 60 mil/min (HR = 1.107, 95% CI: 0.939–1.307, *p* = 0.227) at index hospitalization (Figure 5).

## 4. Discussion

The present study sought to investigate the prevalence and prognostic impact of anemia in a large-scale study of consecutive patients undergoing CA. Concomitant anemia was prevalent in 38.5% of patients undergoing CA and independently associated with the risk of 36-month HF-related rehospitalization, even after multivariable adjustment and propensity score matching. This was the case despite having several surrogate parameters for different anemia etiologies present in our regression analyses, such as BMI (nutrition status), eGFR (kidney-failure-associated anemia), and malignancy (anemia of chronic disease). When further stratifying by hemoglobin levels (per 1 g/dL decrease), patients with Hb 9–<10 g/dL had the highest rates of HF-related rehospitalization. Patients with lower or higher hemoglobin levels experienced lower rates of HF-related rehospitalization. The prognostic value of anemia was especially evident in patients presenting with multivessel disease, LVEF ≥ 35%, and without diabetes mellitus or malignancy at index hospitalization.

Malignancies represent one of the most common causes of anemia, and both malignancy and its treatment are often associated with HF. Because of this, we wanted to control for this variable by including malignancy in both our multivariate analysis and subgroup analysis. While malignancies were more common in anemic patients, malignancies did not significantly influence the risk of HF-related rehospitalization, and there was no significant difference in anemia-induced risk of HF-related rehospitalization in both the malignancy and nonmalignancy subgroups.

When investigating the prognostic impact of anemia, most recent registries have focused on strict inclusion criteria such as STEMI, NSTEMI, or HF, thus analyzing only a subset of patients admitted during daily clinical practice [13,14,15]. The rates of concomitant anemia range from 23 to 71% in these studies, suggesting anemia to be one of the most common noncardiac comorbidities in CAD patients [16,17]. One downside of this approach is that the prognostic value of anemia is always conditional on the cohort-defining diagnosis. This study includes all consecutive patients presenting for CA, with no further exclusion criteria and, as such, allows for a comprehensive comparison of anemia’s prognostic impact across patient subgroups, more independent of the diagnosis on admission. This may provide a more accurate portrayal of the modern patient who is increasingly burdened with cardiac and noncardiac comorbidities due to demographic changes, making risk stratification more difficult.

Many studies investigating the prognostic impact of anemia investigate the risk of all-cause mortality as the primary endpoint. These studies have identified the presence of anemia as an independent predictor of all-cause mortality in patients with various forms of cardiovascular disease, such as HF and ventricular tachyarrhythmias, as well as in the general population [6,16,17]. This result was reproduced by da Silveira et al., even after multivariable adjustment, in a cohort of 310 patients with stable coronary artery disease [18]. When investigating the effect of individual Hb levels on mortality, authors often either stratify anemic Hb levels insufficiently [19], not at all [20], or only investigate anemic Hb levels [21]. All these studies observed a J-/U-shaped relationship between Hb and mortality. Investigating the effects of anemia on mortality in patients with HF, Young et al. included more than 48,000 patients enrolled in the OPTIMIZE-HF Registry to stratify hemoglobin on admission into 1 g/dL groups, ranging from 5 g/dL to 20 g/dL and found a similar J-U/shaped curve [22]. Elevated hemoglobin levels have previously been associated with a worse cardiovascular (CV) prognosis, both within the general population [23] and in CAD patients [24], resulting in the aforementioned U-shaped relationship between hemoglobin levels and CV disease [25,26]. Potential causative factors are high blood viscosity and increased thrombus formation [27]. Brown et al. suggest that the presence of a high hematocrit may indicate the presence of COPD, which would explain the poor CV survival [26].

Scarce data is available on the prognostic impact of anemia on HF-related rehospitalization in both CAD cohorts and unselected cohorts. While not using an unselected patient cohort, Go et al. analyzed data from more than 59,000 patients with HF enrolled in the ANCHOR study and found an increased risk of death and hospitalization for HF in anemic patients, irrespective of HF phenotype [28]. In contrast to this finding, Savarese et al. suggest that a greater risk of all-cause mortality or HF-related hospitalization exists in patients with HFpEF (LVEF ≥ 50%) and HFmrEF (LVEF 41–49%) compared with patients with HFrEF (LVEF < 40%) [15]. Our study’s subgroup analysis suggested a similar phenomenon in a cohort of unselected patients, with an LVEF ≥ 35% (i.e., roughly HFmrEF and HFpEF) being associated with significantly higher anemia-induced risk of HF-related rehospitalization compared with an LVEF < 35%. Savarese et al. propose that anemia may exacerbate HF symptoms more significantly in patients with HFpEF and HFmrEF, leading to increased rates of HF hospitalizations [15]. They explain that a greater symptom burden in patients who have both anemia and HFpEF, combined with the limited effectiveness of HF treatments in HFpEF patients, could explain these findings [15,29]. Our study identified the aforementioned relationship in unselected patients, and thus irrespective of HF-related symptom burden, so another mechanism may be at work.

It is widely known that CAD severity is directly linked to the likelihood, severity, and outcomes of AMI. AMI, in turn, through myocardial ischemia, loss of contractile function, remodeling, and hypertrophy, leads to HF [30,31,32]. Anemia, through hypoxia-induced vasodilation, leads to increased sympathetic activity and cardiac output. If these changes persist, cardiac enlargement and LV hypertrophy ensue [7], resulting in increased oxygen consumption and potentially an aggravation of the already present ischemia. Thus, anemia could interact with CAD to worsen and accelerate AMI and HF. In contradiction to this, Felker et al., in a study including 4951 patients with symptomatic HF undergoing CA, found that that the mortality risk associated with anemia in patients with HF was greatest in patients without CAD and was progressively lower in patients with greater degrees of CAD severity [33]. They suggest that impairment of oxygen delivery is not sufficient a mechanism to explain the association between anemia and mortality in chronic HF. Suggested mechanisms are hemodilution, neurohormonal activation, and inflammation, detrimental cardiorenal interplay, and myocardial remodeling, as well as nutritional deficiency and anabolic/catabolic imbalance [15,34,35,36,37]. Felker et al.’s conclusion is in contrast to the findings of this study, which is that patients with multivessel CAD have an especially higher risk of HF-related rehospitalization if they also have anemia.

While anemia has been shown to be associated with significantly higher mortality rates in patients with AMI by various authors [38,39,40], discrepancies arise when investigating STEMI and NSTEMI separately. For example, data regarding anemia’s influence on mortality in STEMI patients remain inconclusive. While some authors [14,41] suggest that anemia is an independent predictor of mortality after multivariate adjustment, others do not [38,42]. Falluji et al. suggest that factors other than anemia, such as greater left ventricular dysfunction, explain high mortality rates [38]. Regarding patients with NSTEMI, Bayraktarova et al. found significantly higher in-hospital death rates among anemic NSTEMI patients when compared with their nonanemic counterparts [13]. NSTEMI has often been associated with a greater burden of comorbidities such as diabetes and HF [43,44,45,46,47,48], as well as a greater severity and burden of CAD [43,49]. Anemia may interact with these comorbidities through mechanisms like renal insufficiency, hypertension, inflammation, and micro- and macrovascular impairment, exacerbating them [50,51,52]. Moreover, CAD patients seem to have limited baseline coronary reserves due to high oxygen extraction rates within cardiac circulation, a situation anemia would only serve to worsen [53]. Comorbidities and higher CAD severity could explain the higher risk of anemia-associated mortality in patients with NSTEMI.

As demonstrated within the present study, anemia and CAD represent interrelated clinical entities. Even in unselected symptomatic cohorts, and despite improving treatment strategies, the prevalence of anemia is very high, and its impact on 36-month HF-related rehospitalization is significant. As shown above, patients with LVEF ≥ 35% possess a significantly higher anemia-associated risk for HF-associated hospitalization than patients with LVEF < 35%. This, too, is the case for patients with multivessel disease. It is crucial to be aware of this elevated risk and to monitor these patients more closely. In light of the presently occurring demographic change toward a more elderly and comorbid society, timely diagnosis and treatment of anemia as a common comorbidity of CAD is now more important than ever. Tailoring multidisciplinary treatment approaches to address both conditions concurrently is essential, considering the potential impact of anemia on CAD progression and outcomes. This holds true when considering both patient health and quality of life, as well as the economic impact of CAD and anemia. While many recent studies have focused on the potential benefit of treating anemic patients with iron supplementation, this study has found that irrespective of iron levels, hemoglobin levels have significant prognostic relevance. As such, further studies should perhaps focus more on the potential improvements in patient outcomes through hemoglobin-focused therapy.

### Study Limitations

This study has several limitations. With regard to the retrospective and single-center design of this study, results may be influenced by measured and unmeasured confounding factors. To control for these to the best of our ability, propensity score matching was performed. Because patients were identified using Operation and Procedure Classification (OPS) codes, there may be lower documented event rates of prior medical conditions. All endpoints were assessed at our institution only. Additionally, no data regarding long-term all-cause mortality beyond index hospitalization were available. Moreover, no analyses regarding anemia treatment were able to be conducted due to the lack of data on treatment. Finally, while many surrogate parameters for different anemia etiologies were included in our multivariable analysis, the exact etiology of anemia, as well as data concerning iron status, was not available for the present study.

## 5. Conclusions

In consecutive patients undergoing CA, anemia was associated with a significantly increased risk of HF-related rehospitalization at 36 months, even after multivariable adjustment and propensity score matching. Patients with LVEF ≥ 35% and multivessel disease were especially susceptible to anemia-induced HF-related rehospitalization.

## Figures and Tables

**Table 1:** Baseline characteristics.

	Without Propensity Score Matching					With Propensity Matching				
	Non-Anemics (*n* = 4701)		Anemics (*n* = 2944)		*p*-Value	Non-Anemics (*n* = 1914)		Anemics (*n* = 1914)		SMD
**Age**, median (IQR)	66	(56–76)	75	(66–81)	**0.001**	72	(63–80)	74	(65–81)	−10.55%
**Male sex**, *n* (%)	3115	(66.3)	1857	(63.1)	**0.004**	1196	(62.5)	1196	(62.5)	0%
**Body mass index**, kg/m^2^, median (IQR)	27.8	(24.8–31.3)	26.5	(23.8–30.4)	**0.001**	27.7	(24.6–31.2)	26.6	(23.8–30.2)	16.35%
**Cardiovascular risk factors**, *n* (%)										
Arterial hypertension	4093	(87.1)	2419	(82.2)	**0.001**	1794	(93.7)	1778	(92.9)	−3.20%
Diabetes mellitus	1025	(21.8)	922	(31.3)	**0.001**	570	(29.8)	599	(31.3)	3.26%
Hyperlipidemia	1932	(41.1)	788	(26.8)	**0.001**	708	(37.0)	537	(28.1)	−19.08%
**Prior medical history**, *n* (%)										
Congestive HF	300	(6.4)	374	(12.7)	**0.001**	162	(8.5)	222	(11.6)	10.32%
Pacemaker	56	(1.2)	59	(2.0)	**0.004**	28	(1.5)	41	(2.1)	4.51%
COPD	129	(2.7)	168	(5.7)	**0.001**	82	(4.3)	84	(4.4)	0.49%
Liver cirrhosis	33	(0.7)	55	(1.9)	**0.001**	17	(0.9)	33	(1.7)	7.07%
Malignancy	149	(3.2)	295	(10.0)	**0.001**	103	(5.4)	123	(6.4)	4.24%
Stroke	29	(0.6)	31	(1.1)	**0.035**	17	(0.9)	17	(0.9)	0%
**Comorbidities at index hospitalization**, *n* (%)										
Acute coronary Syndrome										
Unstable angina	1502	(32.0)	530	(18.0)	**0.001**	471	(24.6)	422	(22.0)	−6.15%
STEMI	569	(12.1)	341	(11.6)	0.494	185	(9.7)	207	(10.8)	3.63%
NSTEMI	771	(16.4)	615	(20.9)	**0.001**	402	(21.0)	395	(20.6)	−0.99%
Atrial fibrillation	1083	(23.0)	921	(31.3)	**0.001**	558	(29.2)	583	(30.5)	2.84%
Atrial flutter	105	(2.2)	55	(1.9)	0.277	58	(3.0)	44	(2.3)	−4.36%
Acute decompensated heart failure	424	(9.0)	503	(17.1)	**0.001**	277	(14.5)	314	(16.4)	5.26%
Cardiogenic shock	87	(1.9)	234	(7.9)	**0.001**	14	(0.7)	52	(2.7)	15.52%
Atrioventricular block	92	(2.0)	103	(3.5)	**0.001**	39	(2.0)	50	(2.6)	4.00%
Cardiopulmonary resuscitation	161	(3.4)	390	(13.2)	**0.001**	66	(3.4)	78	(4.1)	3.69%
Out-of-hospital	111	(2.4)	273	(9.3)	**0.001**	46	(2.4)	61	(3.2)	4.85%
In-hospital	50	(1.1)	117	(4.0)	**0.001**	20	(1.0)	17	(0.9)	−1.03%
Valvular heart Disease	649	(13.8)	643	(21.8)	**0.001**	397	(20.7)	447	(23.4)	6.52%
Stroke	152	(3.2)	135	(4.6)	**0.002**	53	(2.8)	81	(4.2)	7.62%
**LVEF**, *n* (%)										
>55	2293	(53.0)	1029	(40.8)	**0.001**	946	(49.4)	864	(45.1)	−8.62%
45–55%	962	(22.3)	570	(22.6)		418	(21.8)	461	(24.1)	5.47%
35–44%	509	(11.7)	455	(18.0)		255	(13.3)	320	(16.7)	9.53%
<35%	563	(13.0)	470	(18.6)		295	(15.4)	269	(14.1)	−3.67%
Not documented	374	-	420	-		-	-	-	-	-

COPD, chronic obstructive pulmonary disease; HF, heart failure; LVEF, left ventricular ejection fraction; IQR, interquartile range; (N)STEMI, non-ST-segment elevation myocardial infarction; SMD, standard mean difference. Level of significance *p* ≤ 0.05. Bold type indicates statistical significance.

**Table 2:** Procedural, laboratory, and follow-up data.

	Without Propensity Score Matching					With Propensity Score Matching				
	Non-Anemics (*n* = 4701)		Anemics (*n* = 2944)		*p*-Value	Non-Anemics (*n* = 1914)		Anemics (*n* = 1914)		SMD
**Coronary angiography**, *n* (%)										
No evidence of coronary artery disease	1583	(33.7)	742	(25.2)	**0.001**	524	(27.4)	532	(27.8)	0.89%
1-vessel disease	997	(21.2)	529	(18.0)		370	(19.3)	343	(17.9)	−3.60%
2-vessel disease	952	(20.3)	610	(20.7)		425	(22.2)	396	(20.7)	−3.65%
3-vessel disease	1169	(24.9)	1063	(36.1)		595	(31.1)	643	(33.6)	5.35%
Right coronary artery	1984	(42.2)	1575	(53.5)	**0.001**	943	(49.3)	972	(50.8)	3.00%
Left main trunk	431	(9.2)	410	(13.9)	**0.001**	215	(11.2)	253	(13.2)	6.11%
Left anterior descending	2388	(50.8)	1766	(60.0)	**0.001**	1092	(57.1)	1095	(57.2)	0.20%
Left circumflex	1771	(37.7)	1411	(47.9)	**0.001**	859	(55.1)	877	(54.2)	1.81%
Ramus intermedius	462	(9.8)	386	(13.1)	**0.001**	240	(12.5)	238	(12.4)	−0.30%
CABG	91	(1.9)	134	(4.6)	**0.001**	52	(2.7)	70	(3.7)	5.68%
Chronic total occlusion	344	(7.3)	269	(9.1)	**0.004**	132	(6.9)	147	(7.7)	3.08%
**PCI**, *n* (%)	1953	(41.5)	1335	(45.3)	**0.001**	822	(42.9)	837	(43.7)	1.61%
Right coronary artery	745	(15.8)	530	(18.0)	**0.014**	309	(16.1)	346	(18.1)	5.31%
Left main trunk	154	(3.3)	136	(4.6)	**0.003**	69	(3.6)	83	(4.3)	3.59%
Left anterior descending	1021	(21.7)	691	(23.5)	0.074	428	(22.4)	413	(21.6)	−1.93%
Left circumflex	628	(13.4)	457	(15.5)	**0.008**	303	(15.8)	292	(15.3)	−1.38%
Ramus intermedius	81	(1.7)	56	(1.9)	0.566	38	(2.0)	30	(1.6)	−3.01%
CABG	23	(0.5)	32	(1.1)	**0.002**	14	(0.7)	20	(0.9)	3.27%
**Sent to CABG**, *n* (%)	213	(4.5)	125	(4.2)	0.586	99	(5.2)	97	(5.1)	−0.45%
**Procedural data**										
Number of stents, median (IQR)	2	(1–3)	2	(1–3)	**0.004**	2	(1–3)	2	(1–3)	0%
Stent length, median (IQR)	40	(24–72)	46	(24–80)	**0.015**	43	(24–76)	44	(24–76)	−9.21%
Contrast, median (IQR)	106	(69–181)	126	(75–210)	**0.001**	115	(74–200)	120	(71–200)	−0.97%
**Baseline laboratory values**, median (IQR)										
Sodium, mmol/L	139	(138–141)	139	(137–142)	0.222	140	(138–141)	139	(137–141)	0%
Potassium, mmol/L	3.91	(3.70–4.13)	4.01	(3.76–4.30)	**0.001**	3.95	(3.73–4.15)	3.95	(3.72–4.22)	−8.10%
Calcium, mmol/L	2.24	(2.16–2.32)	2.16	(2.07–2.24)	**0.001**	2.21	(2.13–2.27)	2.17	(2.10–2.25)	23.83%
eGFR, mL/min/1.73 m^2^	73.2	(59.6–86.2)	57.4	(37.9–79.7)	**0.001**	66.1	(52.5–80.0)	61.7	(42.9–83.4)	7.67%
Hemoglobin, g/dL	14.2	(13.4–15.1)	11.0	(9.8–11.9)	**0.001**	14.0	(13.3–14.8)	11.3	(10.1–12.0)	247.21%
WBC count, ×10^9^/L	8.7	(7.1–10.8)	9.4	(7.3–12.4)	**0.001**	8.9	(7.2–10.7)	8.7	(6.8–11.1)	0%
Platelet count, ×10^9^/L	234	(195–276)	237	(185–299)	**0.012**	237	(197–284)	238	(189–296)	−6.08%
HbA1c, %	5.8	(5.5–6.5)	5.9	(5.5–6.9)	**0.004**	6.0	(5.6–6.8)	5.9	(5.4–6.8)	7.97%
LDL-cholesterol, mg/dL	114	(87–143)	89	(66–117)	**0.001**	106	(83–134)	93	(68–120)	34.46%
HDL-cholesterol, mg/dL	43	(36–54)	40	(32–51)	**0.001**	45	(37–55)	41	(33–52)	19.83%
Triglycerides, mg/dL	130	(96–183)	120	(91–169)	**0.001**	132	(98–182)	118	(90–165)	16.82%
C-reactive protein, mg/L	14	(7–39)	60	(19–131)	**0.001**	14	(7–39)	42	(14–100)	−63.47%
Procalcitonin, µg/L	0.15	(0.06–0.65)	0.52	(0.18–2.27)	**0.001**	0.26	(0.11–0.93)	0.29	(0.12–0.97)	5.85%
Albumin, g/L	36.0	(33.5–38.3)	30.5	(26.1–33.9)	**0.001**	35.0	(32.4–37.4)	31.8	(28.3–34.7)	81.28%
INR	1.04	(1.00–1.11)	1.09	(1.02–1.22)	**0.001**	1.04	(0.99–1.13)	1.07	(1.01–1.17)	−13.87%
NT-pro BNP, pg/mL	1064	(249–2964)	3817	(1494–10,246)	**0.001**	1566	(433–4181)	2936	(1158–7013)	−24.73%
Cardiac troponin I, µg/L	0.58	(0.09–5.33)	0.89	(0.15–5.31)	**0.001**	0.46	(0.08–4.30)	0.62	(0.11–4.41)	−7.45%
Creatin Kinase, U/L	130	(84–256)	143	(77–379)	**0.001**	123	(82–225)	126	(72–282)	−7.58%
Creatin Kinase MB, U/L	30	(20–59)	36	(22–73)	**0.001**	33	(23–67)	31	(20–57)	8.40%
**Medication at discharge**, *n* (%)										
ACE-inhibitor	2411	(52.6)	1218	(47.8)	**0.001**	1095	(57.2)	944	(49.3)	−15.88%
ARB	1047	(22.9)	657	(25.8)	**0.005**	438	(22.9)	503	(26.3)	7.90%
Beta-blocker	3202	(69.9)	1845	(72.5)	**0.022**	1437	(75.1)	1435	(75.0)	−0.23%
Aldosterone antagonist	645	(14.1)	423	(16.6)	**0.004**	306	(16.0)	330	(17.2)	3.23%
ARNI	46	(1.0)	32	(1.3)	0.326	20	(1.0)	23	(1.2)	1.92%
SGLT2-inhibitor	253	(5.5)	94	(3.7)	**0.001**	57	(3.0)	79	(4.1)	5.95%
Statin	3425	(74.8)	1842	(72.3)	**0.026**	1451	(75.8)	1399	(73.1)	−6.19%
ASA	2961	(64.6)	1647	(64.7)	0.964	1247	(65.2)	1249	(65.3)	0.21%
P2Y12-inhibitor	2096	(45.8)	1287	(50.5)	**0.001**	924	(48.3)	970	(50.7)	4.80%
OAC	1199	(26.2)	785	(30.8)	**0.001**	630	(32.9)	605	(31.6)	−2.78%
										***p*-values**
**Follow-up data**, median (IQR)										
Hospitalization time	6	(3–9)	10	(5–19)	**0.001**	7	(4–11)	10	(6–18)	**0.001**
ICU time	0	(0–0)	0	(0–1)	**0.001**	0	(0–0)	0	(0–0)	**0.001**
**All-cause mortality, in-hospital**, *n* (%)	120	(2.6)	398	(13.5)	**0.001**	0	(0.0)	0	(0.0)	-
**Patients discharged alive**, *n* (%)	4581	(97.4)	2546	(86.5)		1914	(100.0)	1914	(100.0)	-
**Primary endpoint**, *n* (%)										
HF-related rehospitalization, at 36 months	845	(18.4)	698	(27.4)	**0.001**	499	(26.1)	521	(27.2)	**0.040**
**Secondary endpoints**, *n* (%)										
Acute myocardial infarction, at 36 months	334	(7.3)	214	(8.4)	0.091	175	(9.1)	165	(8.6)	0.919
Coronary revascularization, at 36 months	389	(8.5)	203	(8.0)	0.447	189	(9.9)	157	(8.2)	0.200

ACE, angiotensin-converting enzyme; ARB, angiotensin receptor blocker; ARNI, angiotensin receptor neprilysin inhibitor; ASA, acetylsalicylic acid; CABG, coronary artery bypass grafting; eGFR, estimated glomerular filtration rate; HbA1c, glycated hemoglobin; HDL, high-density lipoprotein; HF, heart failure; ICU, intensive care unit; INR, international normalized ratio; IQR, interquartile range LDL, low-density lipoprotein; NT-pro BNP, amino-terminal pro-B-type natriuretic peptide; OAC, oral anticoagulant; PCI, percutaneous coronary intervention; SGLT2, sodium–glucose-linked transporter 2; SMD; standard mean difference; WBC, white blood cells. Level of significance *p* ≤ 0.05. Bold type indicates statistical significance.

## Data Availability

The datasets used and/or analyzed during the current study are available from the corresponding author upon reasonable request.
